# Perioperative factors associated with delayed graft function in renal transplant patients

**DOI:** 10.1590/2175-8239-JBN-2018-0020

**Published:** 2018-08-13

**Authors:** Milton Halyson Benevides de Freitas, Luciana Cavalcanti Lima, Tania Cursino de Menezes Couceiro, Wilton Bernadino da Silva, João Marcelo de Andrade, Marcio Handerson Benevides de Freitas

**Affiliations:** 1 Instituto de Medicina Integral Professor Fernando Figueira, Recife, PE, Brasil.; 2 Universidade Federal de Pernambuco, Recife, PE, Brasil.

**Keywords:** Kidney Transplantation, Renal Dialysis, Immunosuppressive Agents, Delayed Graft Function, Transplante de Rim, Diálise Renal, Imunossupressores, Função Retardada do Enxerto

## Abstract

**Introduction:**

Successful renal transplant and consequent good graft function depend on a good surgical technique, an anesthetic that ensures the hemodynamic stability of the receiver, and appropriate conditions of graft and recipient. Several factors can interfere with the perfusion of the graft and compromise its viability. The objective of this study was to evaluate perioperative factors associated with delayed graft function (DGF) in renal transplantation patients.

**Methods:**

This is a historical cohort study of patients who underwent renal transplantation between 2011 and 2013. Three hundred and ten transplants were analyzed. DGF was defined as the need for dialysis during the first week post-transplant. Logistic regression with a stepwise technique was used to build statistical models.

**Results:**

Multivariate analysis revealed the following risk factor for DGF: combined anesthesia technique (OR = 3.81, 95%CI, 1.71 to 9.19), a fluid regimen < 50 mL·kg^-1^ (OR = 3.71, 95%CI, 1.68 to 8.61), dialysis for more than 60 months (OR = 4.77, 95%CI, 1.93 to 12.80), basiliximab (OR = 3.34, 95%CI, 1.14 to 10.48), cold ischemia time > 12 hour (OR = 5.26, 95%CI, 2.62 to 11.31), living donor (OR = 0.19, 95%CI, 0.02 to 0.65), and early diuresis (OR = 0.02, 95%CI, 0.008 to 0.059). The accuracy of this model was 92.6%, calculated using the area under the ROC curve. The incidence of DGF in the study population was 76.1%.

**Conclusions:**

Combined anesthesia technique, dialysis for more than 60 months, basiliximab, and cold ischemia time > 12 hours are risk factor for DGF, while liberal fluid regimens and kidneys from living donors are protective factors.

## RESUMO

**Introdução:**

O sucesso do transplante renal e a boa função do enxerto dependem de uma boa técnica cirúrgica, anestesia que assegure a estabilidade hemodinâmica do receptor e condições adequadas de enxerto e receptor. Diversos fatores podem interferir na perfusão do enxerto e comprometer sua viabilidade. O objetivo deste estudo foi avaliar os fatores perioperatórios associados à função retardada do enxerto (FRE) em pacientes transplantados renais.

**Métodos:**

Estudo de coorte histórica em 310 pacientes submetidos a transplante entre 2011 e 2013. A FRE foi definida como a necessidade de diálise durante a primeira semana pós-transplante. Utilizou-se regressão logística e técnica Stepwise para construir modelos estatísticos.

**Resultados:**

A análise multivariada revelou fatores associados à FRE: técnica de anestesia combinada (OR = 3,81,95% CI, 1,71 a 9,19), regime de fluidos < 50 mL.kg^-1^ (OR = 3,71,95% CI, 1,68 a 8,61), diálise por mais de 60 meses (OR = 4,77,95% IC, 1,93 a 12,80), basiliximab (OR = 3,34,95% IC, 1,14 a 10,48), tempo de isquemia fria > 12 horas (OR = 5,26,95 % IC, 2,62 a 11,31), doador vivo (OR = 0,19,95% CI, 0,02 a 0,65) e diurese precoce (OR = 0,02,95% IC, 0,008 a 0,059). A precisão desse modelo é de 92,6%, calculada usando a área sob a curva ROC. A incidência de FRE na população estudada foi de 76,1%.

**Conclusões:**

Técnica combinada de anestesia, diálise por mais de 60 meses, basiliximab e tempo de isquemia fria> 12 horas são fatores de risco para FRE; regimes de fluidos liberais e rins de doadores vivos são protetores.

## INTRODUCTION

Renal transplantation is the treatment of choice for patients with end-stage kidney disease and is associated with better quality of life, a greater cost/benefit ratio, and longer survival.^1^ Proper functioning of the graft is essential for patients to re-establish body homeostasis and thus benefit from receiving the transplant.^2^

Early diuresis is a good marker of successful renal transplantation as it reflects the re-establishment of graft function.^3^ Kidney failure immediately after transplantation, on the other hand, is known as delayed graft function (DGF), which is associated with acute kidney rejection, renal failure and an increased risk of graft loss.^4^ The fluid regimen, living or deceased donor, the anesthetic technique, comorbidities, cold ischemia time (CIT) and the anesthetic drugs used during surgery are some of the factors that may interfere with the perfusion of the graft and compromise its viability.^5^

The aim of this study was to evaluate the perioperative factors associated with delayed graft function in patients who underwent renal transplantation.

## METHODS

Following approval by the hospital ethics committee, this historical cohort study was conducted on patients aged 18-60 years who were scheduled for renal transplantation at our institution in Recife, Brazil between 2011 and 2013. Patients with cardiac disease (ejection fraction < 30), kidney-pancreas transplantation, and kidney retransplantation were excluded from this study.

Hemodynamic instability was considered to be a drop in systolic blood pressure (SBP) of at least 30% compared to the initial blood pressure in the operating room and/or requiring vasoactive drugs for a period equal to or greater than 15 minutes.^6^ Restrictive fluid regimen was defined as the intraoperative hydration < 50 mL·kg^-1^. There are many definitions of DGF in the literature; one of the most well established and adopted in our research was the need for dialysis during the first week post-transplant. Immediate graft function (IGF) is defined as serum creatinine decrease less than 70% of preoperative value in the first week.

Expanded criteria deceased organ donors (ECD) are a source of kidneys that permit more patients to benefit from transplantation. ECD is defined as all deceased donors older than 60 years or donors older than 50 years with two of the following conditions: hypertension, stroke as the cause of death, or preretrieval serum creatinine greater than 1.5 mg/dL^-1^.^7^

Standard monitoring included continuous electrocardiography, heart rate (HR), peripheral oxygen saturation, and noninvasive blood pressure measurement. Induction and maintenance of general anesthesia were performed using sevoflurane in concentrations of 1 to 2% in a mixture of 50% oxygen and 50% air. The most commonly used intravenous anesthetics were fentanyl, propofol and atracurium. Patients underwent balanced general anesthesia (GA) or combined anesthesia (CA), defined by the assistant anesthesiologist depending on hemodynamic stability, comorbidities, and coagulation profile. Regional anesthesia was performed by lumbar epidural injection of bupivacaine 0.125%, follow by general anesthesia. The protocol of immunosuppressive drugs used in our institution is Thymoglobulin and Basiliximab. Thymoglobulin is generally used when the patient has a panel reactive antibody (PRA) > 25% and Basiliximab when the PRA < 25%.

### DATA ANALYSIS

Data were collected from patients' pre-anesthesia evaluation form, intraoperative anesthesia records, and nephrology medical records. The independent variables included anesthesia technique, intraoperative hydration regimen, CIT, duration and type of dialysis, PRA, living or deceased donor, hemodynamic instability, traumatic or non-traumatic death of the donor, receptor comorbidities, type of immunosuppressant used, and weight and age of the donor and receptor. The dependent variable was DGF.

We used the Fisher's exact and Chi-square (χ^2^) tests to compare categorical variables and the Student's *t* and Mann-Whitney tests for mean comparisons (table 1). The independent variables were subjected to logistic regression modeling using a backward stepwise method, according to the Akaike information criterion (AIC).^8^ To evaluate the quality of the regression adjustment, the Hosmer and Lemeshow test was used.^9^ The accuracy and performance of each model were evaluated using the area under the ROC curve.

**Table 1:** Demographic and clinical features of the study population

	IGF (74)	DGF (236)	p-value
**Recipient**			
Gender			0.1767º
Female	27 (36.4%)	73 (30.9%)	
Male	47 (63.5%)	163 (69%)	
Age (years)	42.6 (± 15.1)	45.9 (± 12.6)	0.2962ºº
Weight (kg)	63.5 (± 13.4)	65.2 (± 14.2)	0.3041ºº
BMI (kg.m^-2^) *	22.9 (± 4.2)	24 (± 4.3)	0.4239ºº
Hypertension			0.3424º
No	19 (25.6%)	53 (22.4%)	
Yes	55 (74.4%)	183 (77.6%)	
Diabetes			0.1964º
No	63 (82.8%)	194 (82.2%)	
Yes	11 (17.1%)	42 (17.7%)	
Dialysis type			0.0826º
Peritoneal dialysis	6 (0.8%)	8 (3.3%)	
Hemodialysis	68 (91.2%)	228 (96.7%)	
PRA > 25			0.1556º
No	18 (24.3%)	42 (17.7%)	
Yes	56 (75.6%)	194 (82.2%)	
**Donor**			
Deceased			<0.0001º
No	28 (37.8%)	4 (12.5%)	
Yes	46 (62.1%)	232 (83.4%)	
Age (years)	39.3 (± 15)	42.1 (± 14.5)	0.5368ºº
Cr	1.5 (± 0.8)	2.2 (± 1.9)	0.4495ºº
**Transplantation**			
Cold Ischemia Time (hours)	11.7 (± 11.2)	21.2 (± 7.8)	<0.0001º
Restrictive fluid regimen			<0.0001º
No	43 (58.1%)	64 (27.1%)	
Yes	31 (41.8%)	172 (72.8%)	
Surgical time (min)	153.9 (±31.6)	146.1 (±33.5)	0.3730ºº

SD = Standard deviation (±)

º Chi-square (χ2) tests;

ºº Student's t;

IGF = Immediate graft function;

PRA = panel reactive antibody.

## RESULTS

**Figure 1:**
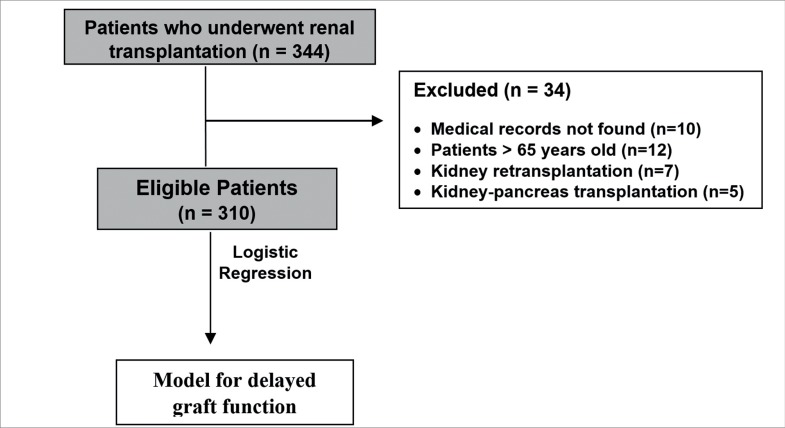
Flowchart of patient inclusion

Three hundred forty-four patients underwent renal transplantation but 34 were excluded from the analysis (Figure 1). The demographic and clinical details of the included patients are shown in Table 1. Both groups were comparable concerning recipient sex, age, and positive versus negative PRA.

Of 310 patients, 67.7% were male, 89% received kidneys from deceased donors, and 112 (36%) received general anesthesia. Upon univariate analysis (Table 1), only the origin of the kidney, restrictive fluid regimen, and the CIT were more prevalent among DGF compared with IGF patients. Hemodynamic instability occurred in 110 patients during the perioperative period, without correlation with any group (*p*-value = 0.48).

ECD organs were present in 109 transplanted patients, 56 in the DGF group (*p*-value = 0.44). Traumatic death occurred in 42.7% (120) of the donors. Stroke was the cause of death of 46.3% (130) and death from others causes was 11%.

The base logistic regression model obtained had the following independent variables: dialysis time, type of anesthesia, immunosuppressive drugs, fluid regimen, early diuresis, and origin of the kidney (Table 2).

**Table 2:** Factors associated with Delayed Graft Function (DGF)

	Estimate of coefficients	*p*-value	OR (95% CI)
RISK FACTORS			
Combined Anesthesia	1.3381	<0.0001*	3.81 (1.71- 9.19)
Restrictive fluid regimen (< 50 mL·kg^-1^)	1.3118	0.00153 *	3.71 (1.68- 8.61)
Immunosuppressant (Basiliximab)	1.2082	0.03201 *	3.34 (1.14- 10.48)
Dialysis >60 months Cold ischemia time (>12h)	1.5641 1.6618	0.00110 * <0.0001*	4.77 (1.93-12.80) 5.26 (2.62 – 11.31)
PROTECTIVE FACTORS			
Living donor	-1.2047	0.03434 *	0.19 (0.02-0.65)
Early diuresis	-3.7312	<0.0001*	0.02 (0.008-0.059)

* *p*<0.05

**Figure 2:**
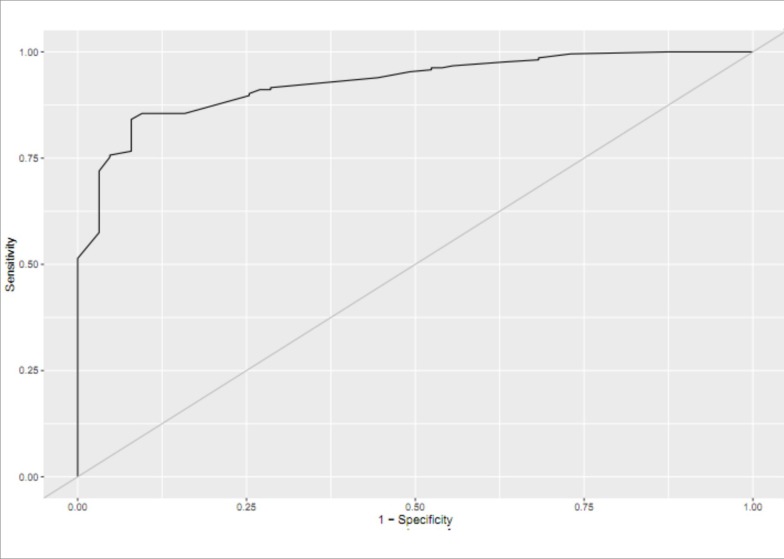
ROC curve showing the sensitivity and false positive rate (1- specificity) of factors associated with Delayed Graft Function (area under the curve: 92.6%).

The incidence of DGF in our study was 76.1%; most of the patients needed dialysis in the first 24 hours (43.1%). Analysis of recipient factors revealed associations of DGF with dialysis time > 60 months (OR = 4.77, 95%CI, 1.93 to 12.80). During the intraoperative time, a restrictive fluid regime (OR = 3.71, 95%CI, 1.68 to 8.61), and the use of combined anesthesia (OR = 3.81, 95%CI, 1.71 to 9.19) were related to DGF. Cold ischemia time > 12 hours (OR = 5.26, 95%CI, 2.62 to 11.31) and basiliximab (OR = 3.34, 95%CI, 1.14 to 10.48) were risk factors to DGF. Among donor factors, living donor was a protective factor against DGF (OR = 0.19, 95%CI, 0.02 to 0.65) and patients with early diuresis had a lower risk of DGF (OR = 0.02, 95%CI, 0.008 to 0.059). To assess the performance of the multiple logistic regression analysis, we calculated the area under ROC curve, which yielded accuracies of 92.6% (Figure 2).

## DISCUSSION

In this retrospective study conducted in 310 kidney transplant patients, we identified several factors related with delayed graft function. Although causality cannot be established, our data suggested associations in agreement with other reports.

DGF is one of the most common complications following renal transplantation and is often associated with acute rejection and increased risk of graft loss. The incidence of DGF in our study was 76.1%, with rates of 0.01% for living donor kidneys and 83.4% for deceased donor kidneys. In a Brazilian multicenter study of 6 transplantation centers, totaling 612 kidney transplants with grafts of deceased donors carried out between 2000 and 2002, revealed an incidence of DGF of 53.9, 62.3, and 51.6% in 2000, 2001, and 2002, respectively.^10^ One center had an incidence of 81.6%, similar to our results. These high rates of DGF of grafts from deceased donors are explained mainly by the difficulty of maintaining hemodynamic stability of the donor, prolonged cold ischemia time, use of ECD kidneys, and the definition for DGF (need for dialysis during the first week post-transplant). Ojo *et al*.^11^ have demonstrated that recipients of ECD kidneys benefit from extra life-years when compared to wait-listed dialysis patients, although they present higher rates of delayed graft function, more acute rejection episodes, and decreased long-term graft function.

Living donor kidneys and early diuresis were identified as protective factors for DGF. The short cold ischemia time and the consequent less pronounced ischemia-reperfusion injury (IRI) produce fewer cytokines and free radicals, which limit the deleterious effects of IRI. A similar association between graft origin and DGF was reported by Ojo *et al.* 12, who conducted a retrospective cohort study of 37,216 patients. In that study, there was a 23% increased risk of DGF for every 6 hours of cold ischemia time in deceased donor kidneys.

DGF rates were also affected by the type of anesthesia used. Combined anesthesia showed a three-fold increased risk of graft dysfunction in our study. This is the first study to correlate anesthetic technique with DGF. The association may be explained by the reduction in catecholamine levels and vasodilatation following neuraxial blockade, which reduces graft perfusion.^13^

A restrictive fluid regimen increased the risk of DGF approximately 4-fold. Kidney perfusion depends linearly on mean arterial pressure, which is influenced by intravascular volume, sympathetic tone, and renal autoregulation.^14^ Due to the denervation during transplantation, self-regulation and sympathetic tone are lost and the renal flow undergoes great influence of volemia.^15^ In a clinical study of 40 patients, Othman *et al*.^14^ evaluated the influence of the hydration regime on hemodynamic stability and early function of the graft and found that patients who received the largest infusion of fluids moments before the renal artery was unclamped presented lower hemodynamic instability and higher levels of systolic blood pressure, medium blood pressure, and central venous pressure.

Duration of dialysis pre-transplantation was also identified as a risk factor for DGF in our study. In a retrospective study of 30,294 kidney transplant patients, Keith *et al.* 16 found that the incidence of DGF was 26.4% higher in patients who received dialysis for more than 72 months.

Basiliximab was identified as risk factor for DGF (OR 3.34, *p*-value = 0.03201). Similarly, in a retrospective cohort study of 327 patients, Chen *et al.* 17 found a higher risk of DGF in the basiliximab group (37.1% versus 26.1%, *p*-value = 0.035). According to Lebranchu *et al.*,^18^ both anti-lymphocyte drugs (Thymoglobulin and Basiliximab) are effective for inducing immunosuppression and are associated with DGF because they release cytokines and induce nephrotoxicity. Thymoglobulin is associated with a lower incidence of graft dysfunction because of its anti-adhesin molecules, which prevent leukocytes from adhering to cell surfaces.^19^

The current study had a few limitations. First, some unmeasured variables may have affected the accuracy of our models. Second, perioperative care practices may be different in other institutions, potentially accounting for the differences in outcomes. Third, the lack of water balance, which would be the ideal variable to characterize peri-operative volume management. Our study has several strengths: this is the first study to examine the effect of anesthesia on DGF, and our end-points are statistically relevant for clinical practice. Future controlled prospective studies should be conducted to further evaluate these results.

## CONCLUSION

In summary, this study showed that liberal fluid regimens, kidneys from living donors, and cold ischemia of less than 12 hours are protective factors for DGF. Dialysis pre-transplantation > 60 months and combined anesthesia are risk factors. Basiliximab is a risk factors for DGF.
